# Predictive Power of the "Trigger Tool" for the detection of adverse events in general surgery: a multicenter observational validation study

**DOI:** 10.1186/s13037-021-00316-3

**Published:** 2022-02-08

**Authors:** Ana Isabel Pérez Zapata, Elías Rodríguez Cuéllar, Marta de la Fuente Bartolomé, Cristina Martín-Arriscado Arroba, María Teresa García Morales, Carmelo Loinaz Segurola, Manuel Giner Nogueras, Ángel Tejido Sánchez, Pedro Ruiz López, Eduardo Ferrero Herrero, The Research Collaboration Group, Antonio Zarco Pleguezuelos, Manuel Romero Simó, Albert Caballero Bouza, David Parés Martinez, Juan Francés Julián Ibáñez, José María Balibrea del Castillo, Xavier Morales Sevillano, Benjamín Díaz-Zorita Aguilar, Lorena Martín Román, Marcos Gomez Ruiz, Tamara Fernández Miguel, Carmen Cagigas Fernandez, Alejandro Moreno Bargueiras, Oscar Cano Valderrama, Daniel Alonso Rivera, María Gutiérrez Samaniego, Manuela Elia Guedea, Elena Córdoba Diaz, Jose Antonio Gracia Solanas, Angela Bañuls Matoses, Ángel Macero, Jose Daniel Sánchez López, María Antonia Vaquero Pérez, Jose Alberto Rojo López, Francisca Lima Pinto, Eneida Bra Insa, Ignacio Rodríguez Prieto, Erlinda Daniela Padilla Zegarra, Mario Franco Chacon, Robert Memba Ikuga, Rosa Jorba Martin, Fernando Alcaide Matas, Paula Troncoso Pereira, Víctor Soria Aledo, Carmen Victoria Pérez Guarinos, Sixto Genzor Rios, Miguel Ángel Dobón Rascón, Sandra Núñez Fernández, Ernesta Valerias Domínguez, Manuel García García, Vanesa Zambrana Campos, Pere Rebasa Cladera, Mariano Artés Caselles, Matías Cea Soriano, Daniel Gambí Pisonero, Santos Jiménez de los Galanes, Maria Dolores Frutos Bernal, Ana Delegido García, Beatriz Gómez Pérez, Carlos Montero Zorrilla, Javier Cortés Climent, Cristina Vallejo Bernad, Ruth Bustamante Mosquera, María Blázquez, Jesús Muriel López, Juan Carlos García Pérez, Juan Ocaña Jiménez, Gloria Paseiro Crespo, Cristina Pardo Martínez, María García Nebreda, José María Fernández Cebrián, Virginia Casanova Durán, Manuel Ferrer Márquez, Javier Aguiló Lucía

**Affiliations:** 1 General and Gastrointestinal Department at 12 de Octubre University Hospital, Avda Córdoba sn, 28041 Madrid, Spain; 2 General and Gastrointestinal Department at Infanta Elena University Hospital, Valdemoro, Spain; 3 Biomedical Research Fundation, 12 de Octubre University Hospital, Madrid, Spain; 4 Madrid Proffesor Surgery Department at Medicine Faculty. Complutense University, San Carlos University Hospital, Madrid, Spain; 5 Urology Department, 12 de Octubre University Hospital, Avda Córdoba sn, 28041 Madrid, Spain

**Keywords:** Adverse event, General surgery, “Trigger Tool”

## Abstract

**Background:**

In spite of the global implementation of standardized surgical safety checklists and evidence-based practices, general surgery remains associated with a high residual risk of preventable perioperative complications and adverse events. This study was designed to validate the hypothesis that a new “Trigger Tool” represents a sensitive predictor of adverse events in general surgery.

**Methods:**

An observational multicenter validation study was performed among 31 hospitals in Spain. The previously described “Trigger Tool” based on 40 specific triggers was applied to validate the predictive power of predicting adverse events in the perioperative care of surgical patients. A prediction model was used by means of a binary logistic regression analysis.

**Results:**

The prevalence of adverse events among a total of 1,132 surgical cases included in this study was 31.53%. The “Trigger Tool” had a sensitivity and specificity of 86.27% and 79.55% respectively for predicting these adverse events. A total of 12 selected triggers of overall 40 triggers were identified for optimizing the predictive power of the “Trigger Tool”.

**Conclusions:**

The “Trigger Tool” has a high predictive capacity for predicting adverse events in surgical procedures. We recommend a revision of the original 40 triggers to 12 selected triggers to optimize the predictive power of this tool, which will have to be validated in future studies.

## Background

Identification of adverse events is relevant for patient safety. The overall rate of adverse events during hospitalization varies from 3% to 17%, of which approximately 50% are deemed preventable [1–3].

Adverse events entail a clinical impact and an increase in resources [4]. The most expensive are surgical, those related to medication and diagnostic delay [5, 6].

Surgical units are the areas with the highest frequency of adverse events [7]. They are related to 1.9% to 3.6% of adverse events in patients admitted to hospital, which represents 46% to 65% of all adverse events in hospitalization [3, 8, 9] .

The most usual methods to detect adverse events (reporting of incidents, record of incidents and clinical-administrative databases) tend to underestimate the actual number of adverse events [10, 11]. Since the publication of the Harvard Medical Practice Study (HMPS) [9], the retrospective methodology to review adverse events has been the most commonly used.

In 2006 the Institute for Healthcare Improvement (IHI) [12] encouraged healthcare systems to implant the Global “Trigger Tool” to measure and monitor injury to the patient. Triggers are specific or global events that are used as key for the selection of medical records that most likely will have a high probability of containing adverse events.

In general surgery the “Trigger Tool” presented sensitivity and specificity of 86.0% and 93.6% respectively. This means it is highly effective to detect adverse events [2, 13].

Development of a specific tool that enables identifying adverse events at low cost, quickly and effectively is of major use in surgery.

The aim of this study is to validate a set of predictive “triggers” for adverse events in patients operated in General surgery and gastrointestinal surgery departments.

## Methods

### Study design

Observational, descriptive study with analytical, retrospective and multicenter components to validate the “Trigger Tool” for detection of adverse events in General surgery and gastrointestinal surgery.

A total of 31 acute care hospitals from the public health system in Spain took part in the study, these hospital are shown in Table 1 (sampling by convenience). 11 of these hospitals were type 1 (under 300 beds), 6 type 2 (301-600 beds) and 14 type 3 (more than 601 beds).

**Table 1:** Spanish Collaborating Hospitals, localization and size by number of beds

Hospital	Lozalization	Size by numer of beds	Type of hospital
General de Alicante Hospital	Alicant	800	3
Barcelona Clinic Hospital	Barcelona	800	3
Lozano Blesa University Hospital	Zaragoza	900	3
Joan XXIII University Hospital.	Tarragona	819	3
Gregorio Marañón University Hospital	Madrid	1150	3
Marques de Valdecillla Univesity Hospital.	Santander	900	3
12 de Octubre University Hospital	Madrid	1368	3
San Carlos University Hospital	Madrid	850	3
Miguel Servet University Hospital	Zaragoza	1400	3
University Hospital	Ourense	869	3
Virgen de la Arrixaca University Hospital	Murcia	900	3
Álava University Hospital	Victoria	800	3
Ramón y Cajal University Hospital.	Madrid	1161	3
Torrecárdenas General Hospital	Almería	821	3
Germans Trias i Pujol Hospital	Badalona	500	2
San Jorge University Hospital	Huesca	312	2
Parc Tauli University Hospital	Sabadell	400	2
Puerta de Hierro University Hospital	Madrid	600	2
Alcorcón University Hospital	Alcorcón	450	2
Morales Messeguer University Hospital	Murcia	320	2
Infanta Sofía University Hospital	San Sebastián de los Reyes	271	1
Infanta Cristina University Hospital	Parla	200	1
Mateu Orfila general Hospital	Menorca	142	1
Francesc de Borja Hospital	Gandía	292	1
Torrejón de Ardoz University Hospital	Torrejón de Ardoz	210	1
Santa Bárbara University Hospital	Puertollano	158	1
Infanta Elena University Hospital	Valdemoro	152	1
Virgen de los Lirios University Hospital	Alcoy	267	1
Infanta Leonor University Hospital	Vallecas	269	1
Tajo University Hospital	Aranjuez	90	1
Lluis Alcanyis	Xátiva	273	1

Type 1 (under 300 beds), type 2 (301-600 beds) type 3 (more than 601 beds)

Patients aged over 18 admitted to General surgery and gastrointestinal surgery from September 1, 2017 to May 31, 2018 who underwent surgery, with full and closed clinical histories and hospital discharge from the same hospital, were included.

Psychiatric, transplanted patients and those referred from other hospitals were excluded.

The sample was calculated randomly according to an estimated probability of 90% for detection of adverse events [2], with an estimated population of 80,000 patients, a 95% confidence interval and precision of 0.02. Sample size was 855 histories distributed among the hospitals taking part. The sample was enlarged to avoid possible case losses and incomplete information.

### Instrumentalization

The “Trigger Tool” was applied to detect adverse events. A total of 40 triggers were included (Table 2).

**Table 2:** Triggers used in the study grouped by modules

Modules	Triggers used in the study
Care module	1. Transfusion of blood or blood derivatives
	2. Cardiorespiratory arrest code
	3. Acute dialysis
	4. Positive blood culture
	5. Radiological test for the study of thrombosis (Unscheduled echo-Doppler during admission, CT angiography)
	6. Sudden decrease in hemoglobin equal or greater than 25%.
	7. Patient fall
	8. Bedsores
	9. Patient detention measures
	10. Readmission 30 days post-discharge
	11. Unscheduled radiology during admission
	12. Infection associated with healthcare
Medication module	1. Positive culture for Clostridium difficile antihistamine
	2. Partial Thromboplastin Time (PTT) over 100 s
	3. INR (International Normalized Ratio) over 6
	4. Glycemia under 50 mg/dL
	5. Increased serum creatinine x 2 compared to basal level
	6. Administration of vitamin K 7. Administration of Flumazenil
	8. Administration of Naloxone.
	9. Administration of Epinephrine.
	10. Administration of anti-emetics
	11. Sudden stoppage of the medication
Surgical module	1. Reintervention in the 30 days post-discharge.
	2. Unscheduled change in procedure or complication of this.
	3. Unscheduled transfer to critical care unit (higher level of care)
	4. Unscheduled intubation or repeat intubation
	5. Intra-operative radiology
	6. Mechanical ventilation greater than 24 hours
	7. Intra-operative administration of Flumazenil, Naloxone or Epinephrine.
	8. Postoperative increase in troponin greater than 1.5 nanograms/mL
	9. Unscheduled injury or removal of an organ
Added based on prior literature and studies	1. Care in the emergency department 30 days post-discharge
	2. Unscheduled invasive procedures during admission (interventional radiology, endoscopy)
	3. Pathological anatomy unrelated to diagnosis
	4. Use of broad spectrum antibiotherapy
	5. Use of Total Parenteral Nutrition.
	6. Prolonged stay in resuscitation after surgery (over 24 hours).

This methodology consists of two phases. An initial screening, where the medical records are reviewed for the identification of triggers. Later, medical records containing any of the triggers (Trigger+) continue to a second part of exhaustive review in order to detect adverse events.

**Fig. 1:**
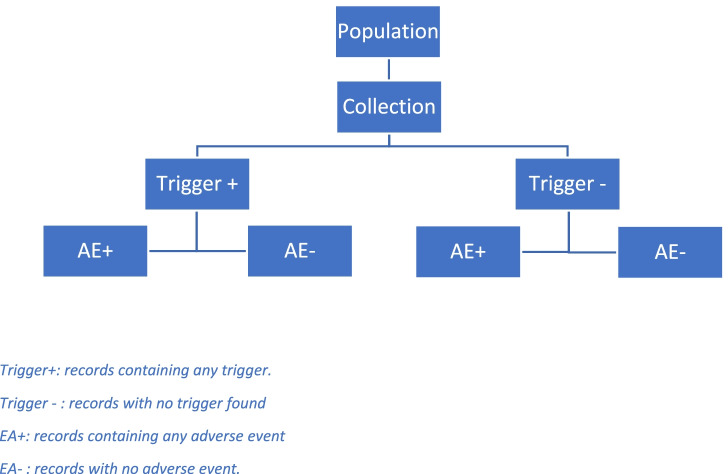
Application of the “Trigger Tool” methodology

To be able to study the predictive power of the tool, those records in which no triggers (Tiggers-) were identified were also reviewed. The application methodology of the tool is summarized in the Fig. 1.

**Fig. 2:**
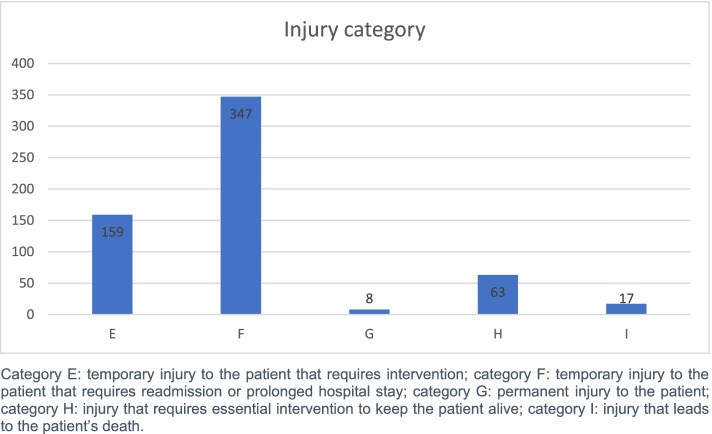
Adverse event by injury category

When the adverse events is identified (EA+), it is defined based on harm category and type of adverse event. For the category of adverse events injury, the “National Coordinating Council for Medication Error Reporting and Prevention” classification [14] (Fig. 2) was used.

A screening guide was published in accordance with criteria on the search for triggers and adverse events and a training video-tutorial. When necessary, the training was completed with an individual tutorial.

### Review process

#### Each center had at least two reviewers.

Clinical histories were reviewed in accordance with the screening guide to identify triggers. Both histories that contained triggers and those that did not were reviewed to search for adverse events. The same information sources and review sequences were used.

Information sources were clinical discharge reports, surgical procedure protocols, medical and nursing clinical course observations from the patient’s admission to 30 days post-discharge, reports of additional tests and prescription of medicines.

Adverse event was considered to be any harmful and unintended event that occurred to the patient as a consequence of the practice of healthcare unrelated to their illness [15].

When an adverse event was detected an injury category was assigned and the degree to which this could have been prevented was assessed. The classification used in the ENEAS study was adapted to determine the preventable nature of the adverse events [16] The study data and variables were recorded in an online database (REDCap). Confidentiality rules were upheld.

This study was approved by the coordinator site’s ethics committee.

#### Statistical analysis

Descriptive analysis by means of mean, median and standard deviation for continuous variables and by means of distribution of frequencies for categoric variables.

The most important variables were compared by means of Mann-Whitney U non-parametric contrast, chi-squared contrast or Fisher test.

To measure the predictive validity of the tool to detect adverse events, diagnostic sensitivity and specificity, in addition to positive predictive value (PPV) and negative predictive value (NPV) were used.

A prediction model was used for the proposed optimization of the tool by means of binary logistic regression. The onset of adverse events and triggers were introduced as dependent and independent variables, respectively. The latter were the statistically significant ones on bivariate analysis.

The model’s results are shown in the form of odds ratio (95% confidence interval [CI]). The model’s discriminatory power was assessed by means of area under the curve (ROC).

The prediction model was repeated for relevant clinical entities such as preventable and severe adverse events and most common procedures.

*P*<0.05 was considered statistically significant for all analyses.

Data were entered by each center’s reviewers into the REDCap database. The statistics program STATA/SE v10.0 was used.

This study has been funded by Instituto de Salud Carlos III through the project "PI17/01374" (Co-funded by European Regional Development Fund/European Social Fund; “A way to make Europe”/"Investing in your future").

The project was approved by the ethics committee of the study coordinating center.

## Results

A total of 1132 cases were recorded. Mean age was 58.15 (18-94). There were 555 (49%) females and 577 (51%) males.

Symptomatic cholelithiasis was the most common diagnosis. This accounted for 13.1% of the total, followed by acute appendicitis (7.2%) followed by inguinal hernia (7.9%), breast neoplasia (5.5%) and eventration (4.9%).

The most common procedures were cholecystectomy (17%), both inguinal and umbilical hernioplasty (13%), appendectomy (7%), eventroplasty (5%) and mastectomy (3%).

Mean stay was 6.5 days (standard deviation 14.32). A total of 73.7% and 26.1% were scheduled and emergency surgical procedures, respectively.

### Behavior of the tool

The tool revealed sensitivity and specificity of 86.27% and 79.55%, respectively. PPV and NPV were 66.52% and 92.48%, respectively. For severe adverse events, sensitivity and specificity were 100% and 26.5%, respectively. For preventable adverse events sensitivity and specificity were 90.3% and 66.9%, respectively.

Table 3 shows the 38 triggers which, after bivariate study, were statistically significant with the onset of adverse events and their onset frequency.

**Table 3:** Trigger and onset of Adverse Event

Trigger	Frequency	*P*
**Broad spectrum antibiotherapy**	171	0.014
**Unscheduled radiology**	162	0.013
**Emergencies 30 days**	112	0.012
**Re-intervention**	80	0.011
**Post-operative TPN**	73	0.011
**Use of Vitamin K**	71	0.001
**Transfusion of blood derivatives**	65	0.013
**Stay in resuscitation >24 h**	63	0.013
**Decrease in Hb >2 g/24 hours**	59	0.01
**Unscheduled ITU transfer**	55	<0.001
**Readmission after 30 days discharge**	54	0.009
**Invasive procedures**	53	0.009
**Transfer to critical care unit**	51	0.009
**Scheduled change in procedure**	38	0.00863
**Basal creatinine x 2**	36	0.008
**Mechanical ventilation over 24 hours**	30	0.00742
**Use of Naloxone**	30	0.00758
**Positive Blood culture**	30	0.00746
**Unscheduled injury of removal of an organ**	24	0.00728
**Reintubation**	21	0.006
**Pathologic anatomy unrelated to diagnosis**	20	0.006
**Unscheduled intubation**	18	0.005
**Sudden stoppage in medication**	13	0.0052
**Cardiorespiratory arrest**	12	0.00479
**Pressure sore**	10	0.004
**Detention measures**	9	0.004
**Intra-operative radiology**	8	0.004
**Acute dialysis**	5	0.003
**Antihistamine**	3	0.002
**Post-operative troponin over 1.5 ng/mL**	3	0.002
**Patient fall**	2	0.001
**Positive stool culture**	1	0.001
**Flumazenil**	1	0.001
**Naloxone**	1	0.001

The triggers that comprised part of the optimized models are shown in Table 4. The model for total adverse events had 12 triggers and its ROC was 83.36 % (CI 81.14%-85.83%). Its predictive capacity is shown in Table 5.

**Table 4:**
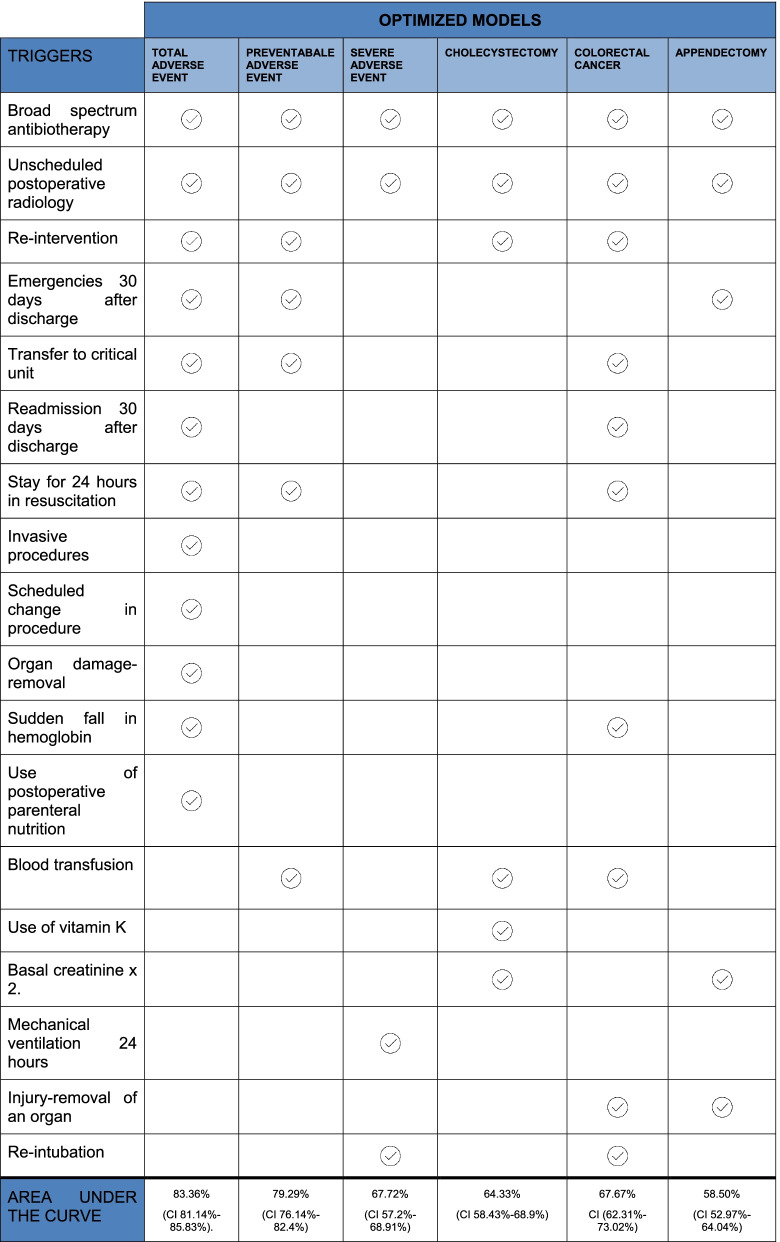
Optimized models and triggers included

existence of trigger

**Table 5:** Predictive capacity of the optimized model for the total adverse event (12 triggers)

	Value	95% confidence interval	
Sensitivity	83.47	79.48	87.47
Specificity	83.25	80.52	85.97
Validity index	83.32	81.09	85.55
PPV	70.12	65.65	74.59
NPV	91.45	89.29	93.61

For preventable adverse events the optimized model led to obtaining sensitivity and specificity of 83.6% and 74.95%, respectively. ROC was 79.29% (CI 76.14%-82.4%).

### Adverse events

The prevalence of adverse events was 31.53% (357 patients). There was a total of 599 AE. A total of 69 patients presented a second adverse event (6.10%) and 28 a third adverse event (2.47%). A total of 16 patients had four or more adverse events (1.41%).

The most commonly observed adverse event were infections (35%). The most common was infection of the surgical site followed by paralytic ileus, intra-abdominal abscess, and anastomotic fistula.

The category of adverse events injury is shown in Graph 1. A total of 34% of adverse events were deemed preventable.

## Discussion

The most important contribution of this study is validation of the “Trigger Tool” in General surgery and gastrointestinal surgery and the proposal for the first time of an optimized model. This enables detecting adverse events more efficiently, which is extremely useful to improve patient safety.

Regarding the different validation methodologies of the “Trigger Tool”, it should be noted that several studies have been performed in other specialties [17–19]. Some works have also published results on optimization of the tool in different areas. This study is, to date, the first on validation of the “Trigger Tool” in General surgery and gastrointestinal surgery and also the first proposed optimized model for this specialty.

One of the methods used to validate the tool was the opinion of experts with Delphi-like surveys [20] on the triggers included in an initial proposal. For some of them the final model included those with a PPV greater than 5% [18, 21]. In others a subsequent study was performed for its validation by means of calculating false negatives in a random sample [19].

Some works report the review of trigger histories. This is the case of the Israeli study [22] on “Trigger Tool” in adverse events related to medication. The optimized model proposed was prepared in accordance with PPV over 10% and the opinion of a panel of experts removing four of the 17 initial triggers. This study only reports adverse events related to medication and the final model is not based on multivariate statistical analysis.

Regarding the predictive capacity of optimized models we found that the study whose results are most similar to this work is the one that uses a similar methodology. In the study by Griffey its model’s area under the curve was 82% with 12 triggers compared to 83.6% in our study.

The PPV of our model (66%) is much higher than that reported in the remaining publications where other methodologies were used with PPV 28.5% [18] and 22.1% [21] where the selection of triggers is not sufficiently accurate.

The studies detected to date do not report specificity or NPV of the tools used as the histories ruled out that did not contain triggers were not reviewed.

Regarding the adverse events identified and described in this study, we highlight the fact that the prevalence detected is greater than that reported in other studies on adverse event [16] but similar to that reported in studies where the trigger methodology was used in 7% to 40% of hospitalized patients [19].

In a scope review performed by Schwendimann et al. it was concluded that half the adverse events were deemed preventable compared to 34% in our study [7]. The variability and subjectivity in regard to the preventability of adverse events was discussed previously. It was recommended not to use this kind of measure [23].

About the severity of adverse events, the most common injury category was F with 58%, followed by category E. These outcomes coincide with those reported in the literature [23, 24].

This work presents certain limitations. A national study required a large number of reviewers and there may be a certain degree of variability. On the other hand, the use of “Trigger Tool” to identify adverse events may not capture all adverse events and information sources may not be reliable. These limitations are part of the IHI’s own methodology.

Despite the mentioned limitations, we consider the multicenter nature of the study, including different types of hospitals within the national health system, to be strictly necessary and a strength that provides power to our work.

In addition, there was a special focus on training reviewers and homogenization of criteria with close tutoring by the research team.

## Conclusions

The “Trigger Tool” proposed in this study is effective to detect adverse events in general surgery and has shown high sensitivity and specificity.

The tool’s optimized model has great predictive capacity with a very considerable reduction in the number of triggers. We recommend a revision of the original “Trigger Tool” (40 “triggers”) to 12 selected triggers to optimize the predictive power of this tool. The results obtained must be validated in future studies.

In any case, the model has proven to be a solid tool for managing patient safety, therefore we recommend its immediate application in the usual clinical practice of general surgery services.

## Data Availability

The study data and variables were recorded in an online database (REDCap). Those are available for testing by reviewers if needed. Confidentiality rules were upheld.

## Abbreviations

IHI: Institute for Healthcare Improvement
PPV: Positive predictive value
NPV: Negative predictive value
ROC: Area under the curve
CI: Confidence interval
